# Extending the pH Stability of Poly(2‐Oxazoline)/Poly(Acrylic Acid) Double‐Network Hydrogels by Including Acrylamide as Comonomer

**DOI:** 10.1002/marc.202500593

**Published:** 2025-11-09

**Authors:** Paola Andrea Benitez‐Duif, Mathusiha Santhirasegaran, Sebastian Weckes, Joerg C. Tiller

**Affiliations:** ^1^ Biomaterials and Polymer Science Department of Bio‐ and Chemical Engineering TU Dortmund University Dortmund Germany

**Keywords:** double‐network hydrogels, hydrogen bonding‐stabilization, pH stability, poly(2‐oxazoline), poly(acrylic acid), poly(acryl amide)

## Abstract

Double‐network hydrogels (DNHs) have outstanding mechanical properties, making them suitable for biomedical applications, such as artificial cartilage. One example of a biocompatible DNH with a mechanical behavior that exactly matches that of natural cartilage is based on poly(2‐methyl‐2‐oxazoline) (PMOx) as primary and poly(acrylic acid) (PAA) as a secondary network, which is stabilized by hydrogen bonds formed between the protonated PAA and the PMOx carbonyl groups. This DNH is shifting the deprotonation of PAA to a pH of 7.6, which makes it applicable in natural tissue. However, implanted hydrogels often encounter varying pH conditions within different tissues or in response to inflammation and infection. Thus, a further shift of the pKa toward more basic conditions is necessary. In this study, the secondary network in the PMOx‐based DNHs was systematically varied by copolymerizing acrylic acid (AA) and acryl amide (AAm) and the mechanical properties were investigated in PBS buffer with varying pH. A ratio of 10 wt.% AAm in the secondary network resulted in a PMOx/P(AA_90_‐co‐AAm_10_) DNH that is not deprotonated even at pH 9 and does not change its dimensions or its cartilage‐like mechanical properties up to this pH.

## Graphical abstract

A double network hydrogel (DNH) based on poly(2‐methyl‐2‐oxazoline) PMOx as primary network and poly (acrylic acid) as secondary network has cartilage‐like properties, which are not affected up to a pH of 7.4. By copolymerizing acrylic acid and acrylamide as a secondary network, a PMOx‐based DNH that is stable up to a pH of 9.0 was obtained.

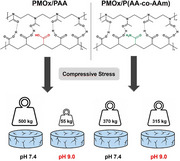

## 1 Introduction

Hydrogels are highly versatile materials with many applications in biomedicine, separation techniques, and soft electronics and robotics [1]. They can be tuned in their properties by either inserting comonomers to insert functional groups and adjust swelling, by inserting polymer segments to add more functionalities, such as gas permeation, e.g., in contact lenses [2], control of protein diffusion [3], and improved mechanical properties [4]. Particularly, the latter is the “Achilles Verse” of hydrogels, which limits their application potential. Different strategies to enhance the strength and toughness of conventional hydrogels have been highlighted, such as incorporating nanocomposites [5], reinforcing agents and interpenetrating two different polymer networks to form a double‐network hydrogel (DNH) [6, 7, 8]. The first double‐network hydrogel system, presented by Gong [9], led to an innovative class of materials characterized by their exceptional mechanical performance. This performance arises from the synergistic combination of two interpenetrating polymer networks, which allows efficient energy dissipation under stress. This unique structural design endows DNHs with superior toughness and durability compared to conventional hydrogels, making them highly suitable for a wide range of applications [10]. One application for such a DNH is the combination of a brittle, fast‐swelling polycation network with a poly(2‐hydroxyethyl acrylate) network to obtain a stable hydrogel with long‐term antimicrobial properties [11].

In the case of the use of DNHs as biomaterial, e.g., artificial cartilage, high strength and stiffness must be contained at the ionic strength and pH of the tissue. Particularly, pH can greatly vary in the tissue during implantation and also in response to inflammation or infection [12, 13]. Therefore, DNH materials that maintain their integrity across these conditions are essential for successful tissue implantation.

Most of the double‐network hydrogel systems with cartilage‐like properties that have been reported are based on at least one polyelectrolyte component, mostly poly(2‐acrylamido‐2‐methylpropane sulfonic acid) (PAMPS) [14, 15, 16], but also poly(acrylate salt) and other polyelectrolytes [17]. The incorporation of ionic groups in the hydrogel network is beneficial for enhancing the water uptake, which is required to obtain a stiff primary network. These groups can also form ionic interactions between the involved polymer networks, which act as sacrificial bonds for the energy dissipation process. The swelling and mechanical performance of hydrogel systems containing polymer networks with ionic groups are highly dependent on salt concentration and on the pH [10, 18, 19].

Besides the often used randomly cross‐linked poly(acrylate)s narrowly distributed hydrophilic polymers such as polyethylene glycol (PEG) and poly(2‐oxazoline) POx can be cross‐linked via their end groups, resulting in more defined hydrophilic networks, which have the potential to act as a primary network for DNHs. We have previously reported on various POx networks purely composed of different POx [20] or as segmented conetworks with acrylates [21, 22]. The combination of a poly(2‐methyl‐2‐oxazoline) (PMOx) network with a poly(acrylic acid) (PAA) as secondary network has resulted in a DNH that perfectly matches the strength and all other mechanical characteristics of articular cartilage independent on salt concentration, at pH up to 7.4, and even in complex media such as egg white [23]. Variation of POx backbone and poly(acrylic acid) derivatives has given deeper insights in the fracture energy dissipation mechanism of such networks, but in no case, the compressive strength of PMOx/PAA could be matched [24]. We have shown that PMOx/PAA is stabilized by hydrogen bonds between PMOx and PAA and loses its compressive strength due to deprotonation in PBS buffer at pH 7.6. The fact that the DNH is not deprotonated at neutral conditions is a remarkable feature of the PMOx/PAA combination, given the fact that the pKa value of PAA is 4.7 [25]. A respective DNH based on PEG and PAA is already losing its strength at pH 5 [26].

However, since slight pH changes above pH 7.4 can result in a loss of mechanical strength, the practical application of PMOx/PAA requires stability at higher pH values. The present study reports on the combination of arylic acid and acrylamide in the secondary network of PMOx‐based DNHs that profoundly improves the stability of the hydrogel against pH changes up to pH 9.

## 2 Results and Discussion

**FIGURE 1:**
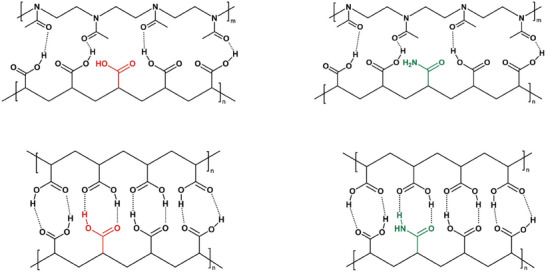
Concept of the structure‐induced potentially higher pH‐stability of PMOx/PAA DNHs by addition of a minor fraction acrylamide.

The high pKa shift of PAA within a PMOx/PAA DNH is presumed to be a result of the strong hydrogen bonds between the protonated carboxylate groups of PAA and the carbonyl function of PMOx. Given the molecular structural differences between PAA and PMOx it becomes clear that not all carboxylate groups have a binding partner (see Figure 1). These groups will eventually be easier, i.e., at lower pH values, deprotonated than the groups that form the hydrogen bonds with the PMOx network. The concept of this study is to exchange the excessive non‐binding carboxylates by another hydrogen donor monomer to obtain an even higher shift in pKa of the PAA. As shown previously, PAAm is suited as DNHs partner for PMOx networks, but results in lower strength due to less strong binding between the two polymers [24].

**FIGURE 2:**
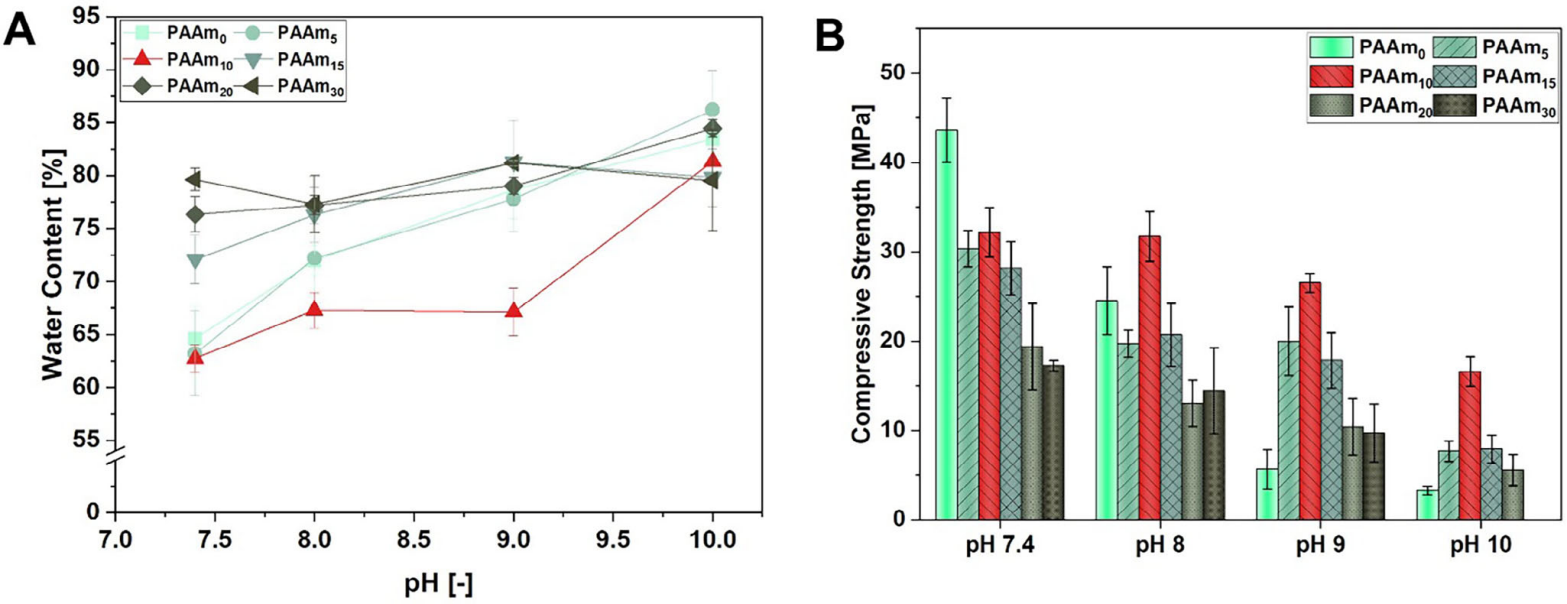
Water content (A) and compressive strength (B) of PMOx/P(AA‐co‐AAm) DNHs with varying compositions swollen in PBS buffer with different pH values at room temperature. All values are expressed as mean ± SD (n = 5).

Therefore, we chose acrylamide as comonomer for PAA to reduce the number of non‐binding carboxic acid groups in the DNH. Given this proposed concept, an optimal ratio of acid and amide groups is expected to obtain increased pH stability. On one hand, a lower number of amide groups will not substitute a sufficient number of carboxic acid groups. On the other hand, a higher number of the amide groups will result in a weaker binding between the PMOx and the poly acrylic network, which will result in an even greater pH sensitivity. A series of PMOx/P(AA‐co‐AAm) DHNs with varying ratios of AA:AAm was prepared according to previously published protocols. Therefore, PMOx with 50 repeating units and two acryl amide end groups was cross‐linked by photopolymerization and the primary PMOx_50_ network was then soaked in the monomer mixture containing TEGMA as cross‐linker and a photoinitiator followed by another photopolymerization step. The resulting DHNs were tested regarding their mechanical properties in water as well as in PBS buffer at different pH values. Prior to investigating the mechanical properties, the water uptake of the DNHs in different media was measured. A change in water uptake automatically results in a change in dimensions, which is problematic, because this makes fitting of implants imprecise and can lead to problems in case of an infection by building pressure or loosening of the implant. As seen in Figure 2A, the water uptake of the investigated DNHs with changing pH of the PBS buffer is strongly dependent on the composition of the secondary network.

The PMOx/PAA DNH is continuously increasing the water uptake from 60 to 80 wt% with increasing pH (Figure 2A). This is due to the fact that higher pH leads to increased deprotonation of the acid groups and an ionic hydrogel takes up more water caused by the repelling of the charged groups in the polymer chains and the increased osmotic pressure. Adding 5 wt.% of acrylamide to the PAA network does not change the swelling characteristics of the resulting DNH. However, the addition of 10 wt.% of AAm to PAA shows a dramatic effect on the pH‐dependent swelling. The PMOx/P(AA‐co‐AAm) DNH has a water content of 62 wt.% in PBS buffer pH 7.4, which stays nearly constant even if the pH in the buffer is increased to 9.0 (water content 67 wt.%). This indicates that there is only a minor degree of deprotonation in this broad pH range. Further increasing the pH to 10 results in a dramatic increase in water uptake to 81 wt.%, which marks the limit of the hydrogels stability against deprotonation. Increasing the AAm content to 15 wt.% leads to a loss in stability of the DNH against deprotonation. Here, the water uptake in PBS pH 7.4 is 72 wt.% already. Further increasing the pH in the buffer affords a higher water uptake to about 81 wt.% in PBS pH 9.0. Thus, the deprotonation resistance of the acid groups is lowered significantly. If the AAm content is increased to 20 and 30 wt.%, respectively, the DNHs reach their maximum degree of swelling already in PBS pH 7.4, indicating that the higher number of amide groups is diminishing the stabilizing effect of the PMOx/PAA DNH against deprotonation.

As shown previously, the deprotonation of the acid groups in PMOx/PAA results in a dramatic loss in compressive strength [23]. In order to investigate this effect for the here presented DNHs, the mechanical properties were investigated in the PBS buffer of different pH. As observed in the mentioned previous study [23], PMOx/PAA loses almost half of its compressive strength when changing the pH in the PBS buffer from 7.4 (43 MPa) to 7.6 (24 MPa) and is again dropping to 17 MPa at pH 8.0 and to 7 MPa at pH 9.0. As shown in Figure 2B, the addition of 5 wt.% AAm to the PAA phase results in a somewhat lower compressive strength at pH 7.4 (28 MPa), but it drops less with increasing pH and affords an almost three times higher compressive strength (20 MPa) at pH 9.0 compared to the PMOx/PAA system. Increasing the AAm content in the PAA network to 10 wt.% results in a PMOx‐based DNH with 31 MPa compressive strength in PBS pH 7.4, which does not significantly change in PBS pH 8.0. The compressive strength of this DNH only slightly drops to 28 MPa in PBS pH 9.0. and has still a remarkable strength of 18 MP in PBS pH 10.0. Thus, the addition of 10 wt.% of AAm to the PAA network is strongly stabilizing the swelling as well as the compressive strength of the respective PMOx‐based DNH against pH changes up to a pH of 9. This indicates that the concept shown in Figure 1 is valid. The observation that PMOx/P(AA_90_‐co‐AAm_10_) has a somewhat lower compressive strength than PMOx/PAA at pH 7.4 is due to its energy dissipation mechanism, which requires a strong binding between the poly(2‐oxazoline) network and the acrylic network, as shown recently [27]. This binding is weakened by the introduced amide groups resulting in a slightly decreased strength. Further increasing the AAm content does result in somewhat stronger compressive strength. This is again due to the fact that the hydrogen bonds formed between PMOx and PAAm and also within the PAA network are weaker, leading to lower strength. The respective PMOx/PAAm has only 12.7 MPa in compressive strength [24]. Also, the pH sensitivity of compressive strength is increasing with higher AAm content in the poly acrylic network of the PMOx‐based DNHs. This can also be explained by the weaker binding between the two polymer networks in the DNHs.

**FIGURE 3:**
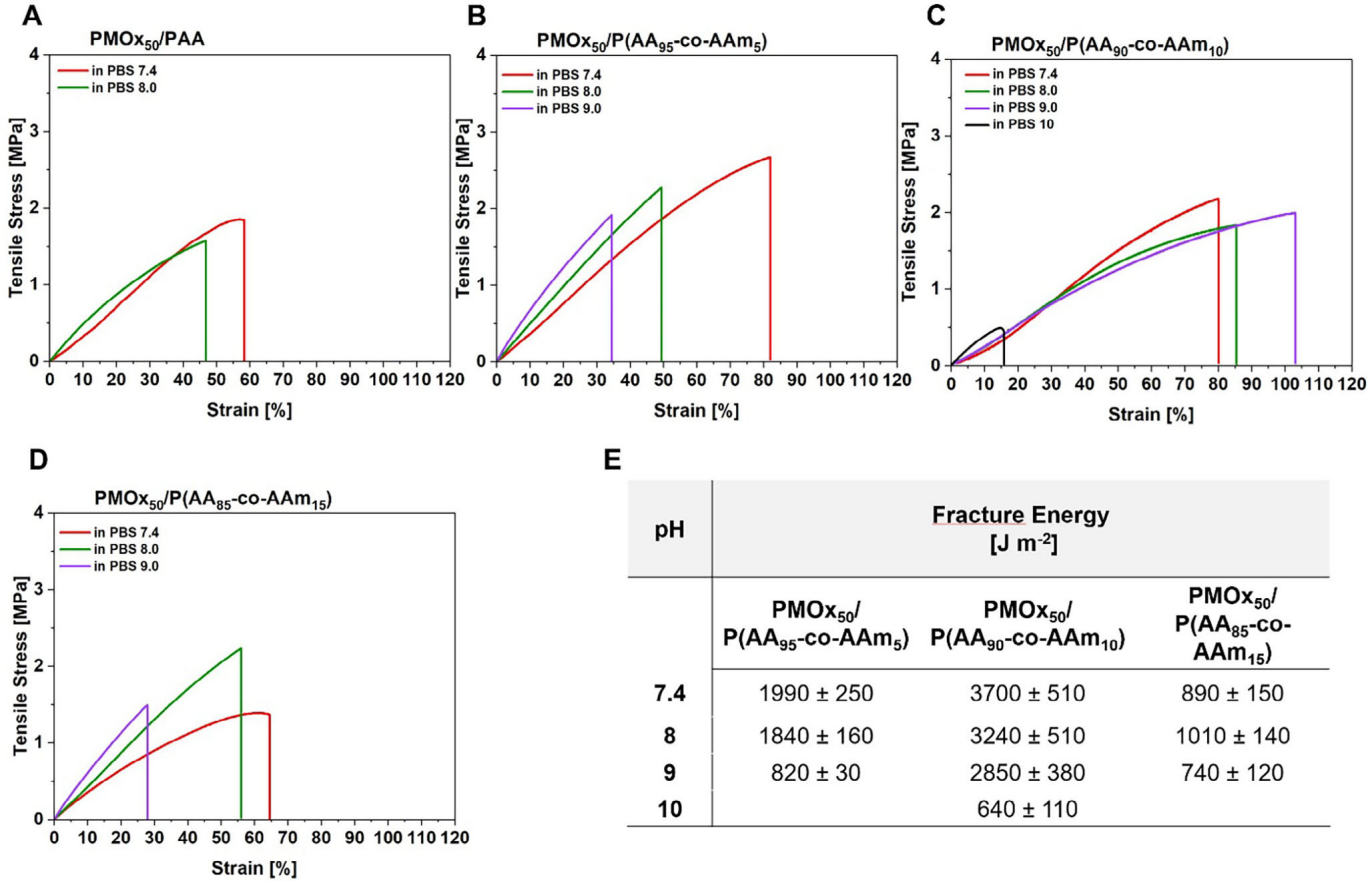
Stress–strain curves of A) PMOx/PAA, B) PMOx/P(AA_95_‐co‐AAm_5_), C) PMOx/P(AA_90_‐co‐AAm_10_), D) PMOx/P(AA_85_‐co‐AAm_15_) and E) fracture energies of different PMOx/P(AA‐co‐AAm) DNHs in PBS buffer with different pH at room temperature The values in E) are expressed as mean ± SD (n = 4).

Taking a look at the tensile toughness and strength of the PMOx/PAA networks with varying AAm content results in a similar picture as the compressive strength. When increasing the pH above 7.4, the PMOx/PAA system without AAm becomes increasingly stiffer, loses its tensile strength, and turns rather brittle (Figure 3A). This DNH cannot be clamped into the tensile tester after swelling in PBS pH 9.0. This effect might be explained by the increased degree of deprotonation, which turns the DNH stiffer due to repulsion of the charge groups, which results in higher water uptake and loss of hydrogen bonding. The addition of 5 wt% AAm to the PAA network results in a DNH with a similar pH dependence as the PMOx/PAA DNH (Figure 3B). Only the relatively low fracture energy of some 2000 J/m^2^ is not significantly changing up to PBS pH 8.0 indicating a somewhat stabilizing effect of the addition of AAm. A different scenario is found for the PMOx/P(AA_90_‐co‐AAm_10_). As seen in Figure 3C, the pH stabilizing effect of AAm is keeping the tensile strength and the strainability of the DNH constant even in PBS pH 9.0. Only, the fracture energy is slightly lowered from 3700 J/m^2^ in PBS pH 7.4 to 2800 J/m^2^ in PBS pH 9.0. The network can be stretched to more than 100% in PBS pH 9 and the tensile strength is still more than 2 MPa as is the case in PBS pH 7.4. Only in PBS pH 10.0, the PMOx/P(AA_90_‐co‐AAm_10_) becomes more brittle and loses its high toughness. Increasing the AAm content in the PAA phase to 15 wt.% leads to a less stretchable, less tough, and more pH sensitive DNH, similar to PMOx/P(AA_95_‐co‐AAm_5_), with the difference that the fracture energy and thus the toughness is significantly lower (1000 kJ/m^2^). This trend continues with adding 20 and 30 wt.% of AAm, respectively, to the PAA phase. Altogether, only the PMOx/P(AA_90_‐co‐AAm_10_) DNH is significantly improving the already high resistance against deprotonation at pH values above the pKa of PAA compared to the respective PMOx/PAA DNH. Our starting hypothesis suggested that this is due to the imperfect binding between the acrylic acid groups and of the PAA network and the carbonyl groups of the PMOx network. The very narrow window of an effective AAm content that allows the stabilization against deprotonation by exchanging the carboxyl groups with neutral weaker binding amide groups supports this hypothesis. In order to check if there is truly no deprotonation of the carboxylic acid groups in PMOx/P(AA_90_‐co‐AAm_10_) at higher pH value, the samples were investigated with ATR‐FT‐IR spectroscopy.

**FIGURE 4:**
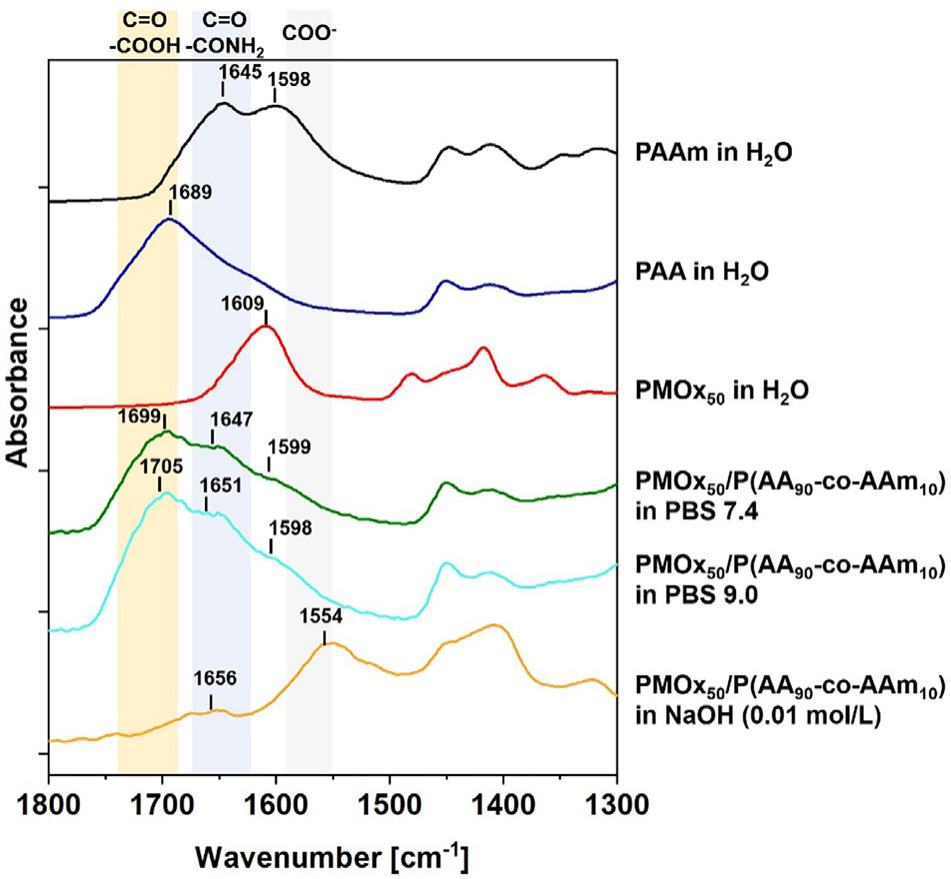
ATR‐FTIR spectra of different hydrogels swollen in different aqueous media.

As seen in the IR spectrum depicted in Figure 4, the DNH PMOx/P(AA_90_‐co‐AAm_10_) swollen in PBS buffer at pH 7.4 shows the signal of the protonated carboxylate groups (1698 cm^−1^) with a slight blue shift at 1714 cm^−1^ and the signal originating from the acryl amide groups at 1651 and 1599 cm^−1^. As known from a previous study [23], the signal of the PMOx carbonyl group undergoes a redshift due to hydrogen formation. Thus, signal is unfortunately overlapping with the signal of acrylamide. The signal expected from the deprotonated carboxylate around 1557 cm^−1^ is missing, indicating no detectable deprotonation at this pH. When shifting the pH in the PBS buffer to pH 9.0, the IR spectrum does not change and also shows no protonation signal. The IR spectrum of the DNH in aqueous sodium hydroxide shows complete deprotonation indicated by the disappearance of the signal for the protonated carboxylate groups at around 1700 cm^−1^ and the appearance of a new signal at 1554 cm^−1^, which can be attributed to completely deprotonated PAA [28]. The latter signal is already seen, when adding the PMOx/PAA DNH without acrylamide to PBS at pH 7.6 [23]. Thus, the IR spectra clearly show that the addition of 10 wt.% AAm to the PMOx/PAA network protects the acid groups from deprotonation up to a pH of 9.0. Although there is no detectable deprotonation in the FTIR, the slight loss of compressive strength from 31 MPa at pH 7.4 to 28 MPa at pH 9.0 and the somewhat lowered toughness at pH 9.0 suggests that there might be a minor, possibly local deprotonation, which has a small influence on the mechanical properties of this DNH. Altogether, the protection against deprotonation in the PBS buffer is most likely the reason for retaining the excellent mechanical properties of PMOx/P(AA_90_‐co‐AAm_10_) DNH even under basic conditions.

## 3 Conclusions

The goal of the present study was to stabilize DNHs based on PMOx as primary and PAA as secondary network against deprotonation in PBS at pH values above 7.4 in order to avoid undesired change of swelling and loss of mechanical properties under basic conditions. The hypothesis was that not all of the acrylic acid groups in PAA bind to the carbonyl groups of PMOx due to mismatching molecular structures. These non‐binding carbolic acid groups are then prone to deprotonation, resulting in the loss of strength and toughness. To avoid deprotonation of these few free carboxylic acid groups they were substituted by amide functions by adding minor amounts of acryl amide to the acrylic acid solution that is used to form the secondary network within the primary PMOx network. The results show that a very narrow range of acrylamide concentration peaking at 10 wt.% in the AA monomer is suited to stabilize the high compressive strength and tensile toughness of the DHNs to up to pH 9.0. This greatly improves the applicability of the DNHs in biological applications. It further shows how much the mechanical properties of POx‐based non‐ionic DNHs depend on the exact matching between the two interpenetrating polymer phases. The present design now allows for biomedical testing of the potential cartilage substitute and can be applied toward novel non‐ionic DNHs with exceptional mechanical properties that might also be used in bendable electronics or membrane technologies such as pressure‐resistant separation membranes.

## 4 Experimental Section/Methods

### 4.1 Materials

Chloroform (Fischer Scientific) was distilled from activated aluminum oxide (Merck) under reduced pressure and under argon atmosphere stored. The monomers 2‐methyl‐2‐oxazoline (MOx) was obtained from Acros Organics, these were distilled over CaH_2_ (Acros Organics). *Trans*‐1,4‐dibromo‐2‐butene (DBB, Acros Organics) was recrystallized twice from n‐heptane (Fischer Scientific). N,N‐Dimethylaminopropylmethacrylamide (DMAP‐MAA, Sigma–Aldrich) was distilled under reduced pressure. All distilled chemicals were stored under argon atmosphere and at −20°C. Commercial phosphate buffered saline (Sigma–Aldrich) was adjusted in its pH with aqueous sodium hydroxide (0.1 m). Acrylic acid (AA, 98% extra pure, Acros Organics), Acrylamide (AAm, VWR), Irgacure 2959 (IG2959, >98%, TCI‐Europe), Irgacure 651 (IG651, Ciba Specialty Chemicals), Tetraethylene glycol dimethacrylate, (TEGDMA, Sigma–Aldrich), methanol (Fischer Scientific), diethyl ether (Honeywell Riedel‐de‐Haën).

### 4.2 Synthesis of Poly(2‐Metyl‐2‐Oxazoline) Macromonomer with DMAP‐MAA End Groups

The synthesis of telechelic PMOx was performed according to previously works of Tiller et al. [23, 29]. The cationic ring opening polymerization of 2‐methyl‐2‐oxazoline (MOx, 5 mL) with DBB (0.25 g) as initiator was carried out in dry chloroform (20 mL) under argon atmosphere in an industrial microwave reactor. After a polymerization time of 2.5 h at 105°C, the living ends of the resulting polymer were terminated with 2.1 mL of N,N‐Dimethylaminopropylmethacrylamide (DMAP‐MAA) in a tenfold molar excess (with respect to the initiator) at 49°C for 72 h. The resulting polymeric product was purified by precipitating the polymer three times in ice‐cold diethyl ether and subsequent dialysis against a mixture of methanol/H_2_O (1:1) using benzoylated cellulose membranes (1000 MWCO). The methanol was removed under reduced pressure and the remaining aqueous solution was dried by lyophilization.

### 4.3 Fabrication of the PMOx‐Based Double‐Network Hydrogels

The double networks hydrogels were synthesized via the two‐step polymerization method, which was first developed by Gong et al. [9]. The primary hydrogel network was prepared from an aqueous solution with 34 wt.% macromonomer PMOx with DMAP‐MAA end groups and photoinitiator IG2959 (4.6 wt.% respect to the macromonomer). This precursor solution (700–1000 µL) was placed between two microscope slides, which were previously coated with poly(propylene)‐tape and separated by 1.05 mm thick spacers for the compression‐tested samples. Spacers with 0.35 mm thickness were used for tensile‐tested hydrogels, so that these could be fixed between the clamps of the tensile tester. Then, it was reacted in a UV curing chamber (Emmi‐Classic automatic, 36 W, λ = 340 nm) for 36 min (switching each side every 2 min). Afterward the resulting network was soaked in DI water over night, in order to remove the unreacted macromonomers and impurities.

To incorporate the secondary co‐network, the primary network was carefully removed from water and immersed in the different solutions based on acrylic acid (AA) and acrylamide (AAm) over night. The co‐acrylate solutions contain photoinitiator IG651 (0.24 wt.% with respect to the total acrylate amount) and tetraethylene glycol dimethacrylate (TEGDMA) as cross‐linking agent (0.19 wt% with respect to the weight of the monomers. Afterward, the swollen hydrogel was placed between the glass plates and cured under a UV source for 20 min (switching each side every 2 min). The final PMOx/P(AA‐co‐AAm) double network hydrogel was removed from the glass plates and washed thoroughly with water for at least 48 h. All washed DN hydrogels were dried at room temperature and given in the corresponding swelling media (PBS pH 7.4, 8.0, 9.0, and 10.0, approx. up to 100 mg dry DNH/L) at least 48 h before the measurements.

### 4.4 Determination of the Water Content

Between 3 and 4 dry hydrogel samples with 6 mm diameter were immersed in deionized water until equilibrium was reached, which corresponds to a swelling time of at least 48 h. Subsequently, the swelled hydrogels were dried under vacuum at 50°C for 48 h. To determinate the water content (WC), the wet (*m*_g_) and dry (after swelling, *m*_d_) weights of the DN hydrogels were measured and calculated as follows:

(1) $$\mathrm{WC}\;\left(\mathrm{wt}.\%\right)=\frac{m_{g}-m_{d}}{m_{g}}\times 100$$

### 4.5 Determination of the Polyacrylate Content in the Double Network

The content of the secondary network in the double network was calculated from the dry weight of the POx‐based primary network (m_1N_) before its swelling in the acrylate solution and the dry weight of the resulting DNH (m_DN_) after the washed process:

(2) $$\mathrm{PA}\;\left(\%\right)=\frac{m_{\mathrm{DN}}-m_{1N}}{m_{\mathrm{DN}}}\times 100$$

### 4.6 Tensile Mechanical Test

The tensile stress–strain‐curves were recorded at room temperature using an Instron 3340 tensile tester with a cell with load 1 kN. At least four swelled dog‐bone tensile samples of different double networks with average dimensions of 4.55 mm × 29.9 mm × 0.76 mm (width x length x thickness) were fixed between the clamps with the help of sandpaper resulting in effective lengths (l_o_) between 15 and 18 mm. The experiment was performed at a crosshead speed of 5% min^−1^ until failure of the sample. During the whole experiment, the samples were kept hydrated with the corresponding swelling medium using a spray bottle.

The determination of the fracture of energy was determined based on previous works of Tiller et al. [5]. Three additional samples, with the same dimensions as mentioned above, were notched in the center. The notch length corresponds to one‐third of the sample´s width. The tensile test of the notched samples was performed until failure as described above. This experiment allows to determinate the strain at which the notch turns into a running crack. The corresponding fracture energy (Γ, in J m^−2^) of the samples was calculated with l_0_ as the length of the sample between the clamps and the area under the stress–strain curve of the un‐notched sample until *ε*_c_ (breaking strain of the notched sample) as follows:

(3) $$\Gamma =l_{o}\;\times \int_{0}^{\varepsilon_{c}}\sigma \left(\varepsilon \right)d\varepsilon$$

### 4.7 Compressive Mechanical Test

The compressive behavior of the DNHs was evaluated with an Instron 5967 tester with a cell load of 30 kN at room temperature. Five circular samples with a diameter of 12 mm and thickness between 1.8 and 2.2 mm were tested. The sample was placed in the compression cell, covered with swelling medium and compressed with an initial preload of 1N. All samples were compressed with a compressive strain rate of 0.5 mm min^−1^ until failure.

### 4.8 Statistical Analysis

All values were expressed as mean ± standard deviation (SD). The results were analyzed statistically using a one‐way ANOVA, followed by a Tukey post‐hoc test. In all cases, the significance was set at *p* ≤ 0.05. Statistical analysis was carried out using OriginPro 2020b Software.

## Author Contributions

J.C.T. and P.A.B‐D. designed the study, interpreted the results, and wrote the manuscript. P.A.B‐D and M.S prepared the hydrogels, performed the mechanical tests and swelling experiments, S.E. recorded the FTIR‐spectra and wrote and interpreted them in the manuscript.

## Conflicts of Interest

The authors declare no conflicts of interest.

## Data Availability

The data that support the findings of this study are available from the corresponding author upon reasonable request.
